# Angiotensin II Infusion Leads to Aortic Dissection in LRP8 Deficient Mice

**DOI:** 10.3390/ijms21144916

**Published:** 2020-07-12

**Authors:** Jeremy Lagrange, Stefanie Finger, Sabine Kossmann, Venkata Garlapati, Wolfram Ruf, Philip Wenzel

**Affiliations:** 1 Center for Thrombosis and Hemostasis, University Medical Center Mainz, 55131 Mainz, Germany; jeremy.lagrange@inserm.fr (J.L.); Stefanie.Finger@unimedizin-mainz.de (S.F.); sabine.kossmann@yahoo.com (S.K.); Garlapati.VenkataSubbaiah@unimedizin-mainz.de (V.G.); ruf@uni-mainz.de (W.R.); 2 INSERM U1116, DCAC (Acute and Chronic Cardiovascular Deficiency), Université de Lorraine, 54500 Nancy, France; 3 Center for Cardiology–Cardiology I, University Medical Center Mainz, 55131 Mainz, Germany; 4 The Heart Research Institute, 7 Eliza Street, 2042 Newtown, Australia; 5 DZHK (German Center for Cardiovascular Research), Partner Site Rhine Main, University Medical Center Mainz, 55131 Mainz, Germany; 6 Department of Immunology and Microbial Science, Scripps Research, La Jolla, CA 92037, USA

**Keywords:** low-density lipoprotein receptor-related protein 8, angiotensin II, aortic dissection

## Abstract

Myeloid cells are crucial for the development of vascular inflammation. Low-density lipoprotein receptor-related protein 8 (LRP8) or Apolipoprotein E receptor 2 (ApoER2), is expressed by macrophages, endothelial cells and platelets and has been implicated in the development of cardiovascular diseases. Our aim was to evaluate the role of LRP8, in particular from immune cells, in the development of vascular inflammation. Methods. LRP8^+/+^ and LRP8^−/−^ mice (on B6;129S background) were infused with angiotensin II (AngII, 1 mg/kg/day for 7 to 28 day) using osmotic minipumps. Blood pressure was recorded using tail cuff measurements. Vascular reactivity was assessed in isolated aortic segments. Leukocyte activation and infiltration were assessed by flow cytometry of aortic tissue and intravital videomicroscopy imaging. Histological analysis of aortic sections was conducted using sirius red staining. Results. AngII infusion worsened endothelial-dependent vascular relaxation and immune cells rolling and adherence to the carotid artery in both LRP8^+/+^ as well as LRP8^−/−^ mice. However, only LRP8^−/−^ mice demonstrated a drastically increased mortality rate in response to AngII due to aortic dissection. Bone marrow transplantation revealed that chimeras with LRP8 deficient myeloid cells phenocopied LRP8^−/−^ mice. Conclusion. AngII-infused LRP8 deficient mice could be a useful animal model to study aortic dissection reflecting the lethality of this disease in humans.

## 1. Introduction

Low-density lipoprotein receptor-related protein 8 (LRP8) or apolipoprotein E receptor 2 (ApoER2) is a member of the low density lipoprotein receptor family and is implicated in premature coronary disease, as well as myocardial infarction [1,2]. Immune cells are important for the development of vascular inflammation, hypertension and atherosclerosis [3]. LRP8 is expressed by macrophages and its absence increases lipid accumulation and lesion progression in atherosclerosis [4].

LRP8 is related to pro-thrombotic diseases such as antiphospholipid syndrome in which it mediates signaling of β2 glycoprotein I together with platelet glycoprotein (GP) Ibα [5]. While overactivity of platelets is an accepted risk factor for vascular disease, few studies have also described the presence of platelet abnormalities as a link to the incidence of vascular inflammation and hypertension [6]. We found that monocyte activation and platelet receptor glycoprotein Ib alpha (GPIbα) participate in a local thrombin amplification, though coagulation factor XI (FXI), promoting the development of vascular inflammation and hypertension [7,8].

LRP8 is able to bind FXI [9] and can mediate activated protein C signaling in endothelial cells and myeloid cells [10]. Moreover, mice lacking LRP8 displayed reduced platelet activation in response to either ADP or thrombin [11]. Sequence analysis of cytoplasmic LRP8 has uncovered several peptide motifs with potential importance for cellular signaling [12], and the complex formed by GPIbα and LRP8 was described to be required to increase platelet aggregation [13].

Acute depletion of platelets with anti-GPIbα immediately blocks leukocyte adhesion to the vascular endothelium. Continuous depletion of platelets protected Angiotensin II (AngII)-infused mice from vascular endothelial dysfunction and oxidative stress. This phenotype was recapitulated in mice with a defective GPIbα [8]. Whether monocytic LRP8 is important in this phenotype is unknown. Furthermore, little is known about other possible roles of LPR8 and related pathways in the development of vascular diseases, such as aneurysm formation or aortic dissection. In patients with aortic dissection independent of the presence of an aneurysm, lipoprotein(a) was elevated compared to control subjects [14], but to date no direct relations between LRP8 genetic variants and aneurysm or aortic dissection have been reported.

Thus, we aimed to explore whether LPR8 expressed by vascular and immune cells has a role in AngII-induced vascular inflammation and dysfunction. Unexpectedly, we observed the formation of aortic dissection in LRP8 deficient mice infused with AngII.

## 2. Results

### 2.1. AngII Induces Vascular Dysfunction and Immune Cell Infiltration in LRP8^+/+^ and LRP8^−/−^ Mice

**Figure 1:**
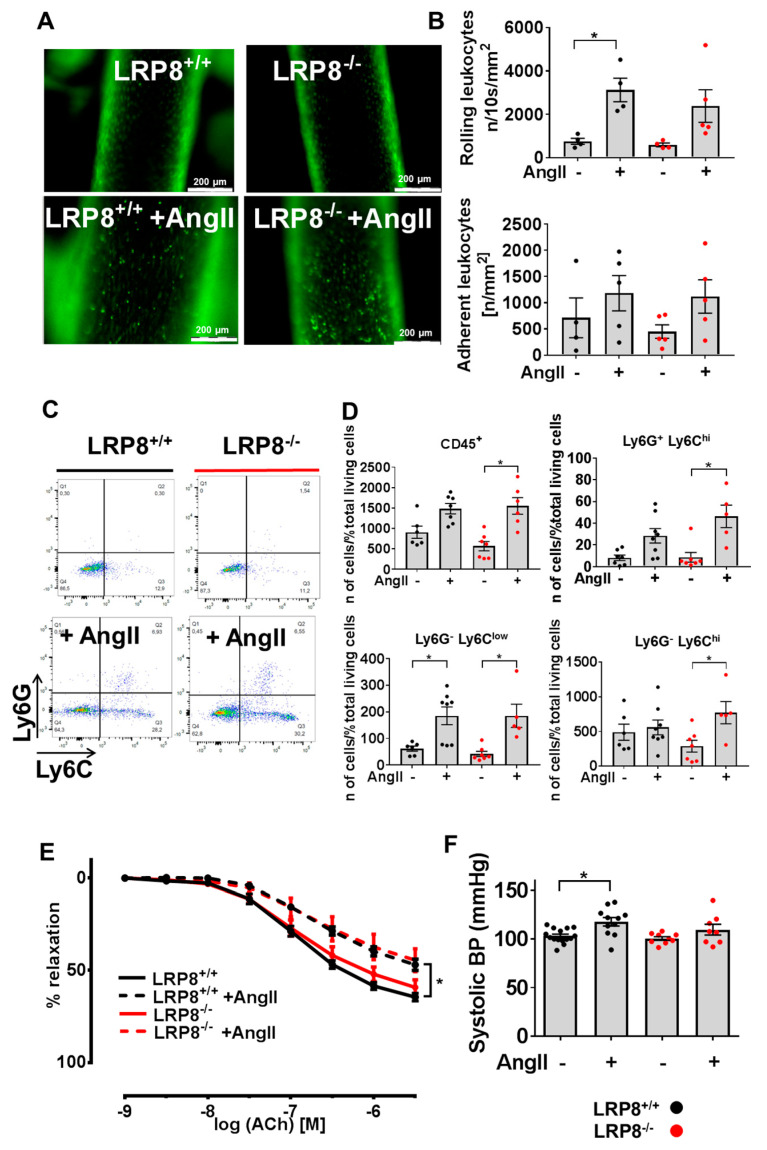
Vascular function and immune cell infiltration in LRP8^+/+^ and LRP8^−/−^ mice in response to AngII. LRP8^+/+^ and LRP8^−/−^ mice were infused with AngII (1 mg/kg/d for 7 d) vs. sham treatment. (**A**) Epifluorescence intravital epifluorescence video microscopy (IVM) of endothelial adherent and rolling leukocytes in the common carotid artery. Nucleated cells were visualized with acridine orange (green fluorescence) (scale bar 200 µm). (**B**) Quantification of adherent and rolling leukocytes. Cell recruitment was quantified in four fields of view (100 × 150 μm) per carotid artery (8 measurements per mouse). Adherent cells were defined in each vessel segment as cells that did not move or detach from the endothelial lining within an observation period of 10 s and presented per mm^2^. One dot corresponds to the mean of 8 measurements in one animal. *n* = 4–5 animals/group. Data are presented as mean ± SEM; * *p* < 0.05; vs. sham treatment of the same strain. One-way ANOVA and Bonferroni’s multiple comparison test. (**C**,**D**) Flow cytometry of aortic lysates. (**C**) Representative original plots. (**D**) absolute numbers of viable CD45^+^ , CD45^+^ CD11b^+^ Ly6G^+^ Ly6C^−^NK1.1^−^, CD45^+^ CD11b^+^ Ly6G^−^Ly6C^low^NK1.1^−^ and CD45^+^ CD11b^+^ Ly6G^−^Ly6C^hi^NK1.1^−^ cells. Results are expressed as the percentage of positive cells per total living cells. One dot corresponds to one aorta of one animal. *n* = 6–8 animals/group. Data are presented as mean ± SEM; * *p* < 0.05; vs. sham treatment of the same strain. One-way ANOVA and Bonferroni’s multiple comparison test. (**E**) Concentration—relaxation curves in response to Acetylcholine (ACh) (endothelium dependent) of isolated aortic segments. One dot corresponds to one aortic ring of one animal. *n* = 5 animals/group. Data are presented as mean ± SEM; * *p* < 0.05; vs. sham treatment of the same strain. Two-way ANOVA and Dunn’s multiple comparison test. (**F**) Systolic blood pressure after one week of AngII-infusion or sham treatment. *n* = 8–14 animals/group. Data are presented as mean ± SEM; * *p* < 0.05; vs. sham treatment of the same strain; one-way ANOVA and Bonferroni’s multiple comparison test.

Mice were infused with AngII for 7 days. AngII infusion increased rolling and adhesion of immune cells on the endothelium of carotid arteries in both LRP8^+/+^ and LRP8^−/−^ mice to the same extent (Figure 1A,B). Accumulation of inflammatory cells was assessed by flow cytometry in aortas of AngII-infused mice. Comparable results between LRP8^+/+^ and LRP8^−/−^ mice were observed for Ly6C^low^ monocytes, while Ly6C^hi^ monocytes and Ly6G^+^ neutrophils were significantly up-regulated only in the LRP8^−/−^ +AngII group (Figure 1C,D). Vascular relaxation studies revealed similar impairment of acetylcholine (ACh)-dependent relaxation in aortas of both LRP8^+/+^ and LRP8^−/−^ mice, following AngII infusion (Figure 1E). Systolic blood pressure was significantly increased in controls, while AngII-infused LPR8 deficient mice only presented a tendency to increase, with lower absolute values than AngII-infused controls (Figure 1F). Blood count was similar in LRP8^+/+^ and LRP8^−/−^ mice before and after AngII infusion (Table 1).

### 2.2. LRP8 Deficient Mice Infused with AngII Develop Aortic Dissections

**Figure 2:**
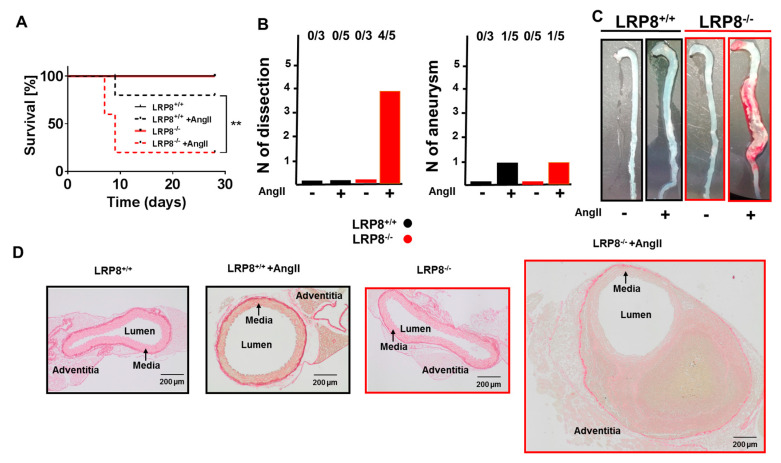
Formation of aortic dissections in LRP8^−/−^ mice in response to AngII. LRP8^+/+^ and LRP8^−/−^ mice were infused with AngII (1 mg/kg/d for 7day) vs. sham treatment. (**A**) Survival curves during 28 days of AngII infusion. *n* = 3–5 animals/group (*n* = 3 in control groups and *n* = 5 in AngII infused groups). ** *p* < 0.01; LRP8^+/+^ + AngII *vs.* LRP8^−/−^ +AngII. Kaplan—Meier curves were compared using a log-rank test. (**B**) Number of aortic dissection and aneurysm formations in LRP8 deficient and control mice infused with AngII. (**C**) Representative images of isolated aortas in control mice and after AngII infusion. (**D**) Representative images of sirius red staining of aortic sections (scale bar 200 µm).

Intriguingly, we noticed that more LRP8^−/−^ mice than LRP8^+/+^ mice died during the 7 days of AngII infusion. When assessing mortality more thoroughly, we noticed that after 28 days of AngII infusion, four out of five LRP8 deficient mice died (Figure 2A). Macroscopic inspections of aortas from AngII infused mice revealed massive aortic dissections in three of the LRP8^−/−^ mice that died prematurely, and an aneurysm in one mouse that died in both the LRP8^+/+^ and LRP8^−/−^ group. We confirmed the presence of dissections in histology by the presence of intravascular hemorrhages also in the aorta of the surviving LRP8^−/−^ mouse infused with AngII, revealing that four out of five LRP8^−/−^ + AngII mice had developed aortic dissections (Figure 2B–D).

### 2.3. AngII-Induced Aortic Dissections are Driven by LRP8 Deficient Bone Marrow Derived Cells

**Figure 3:**
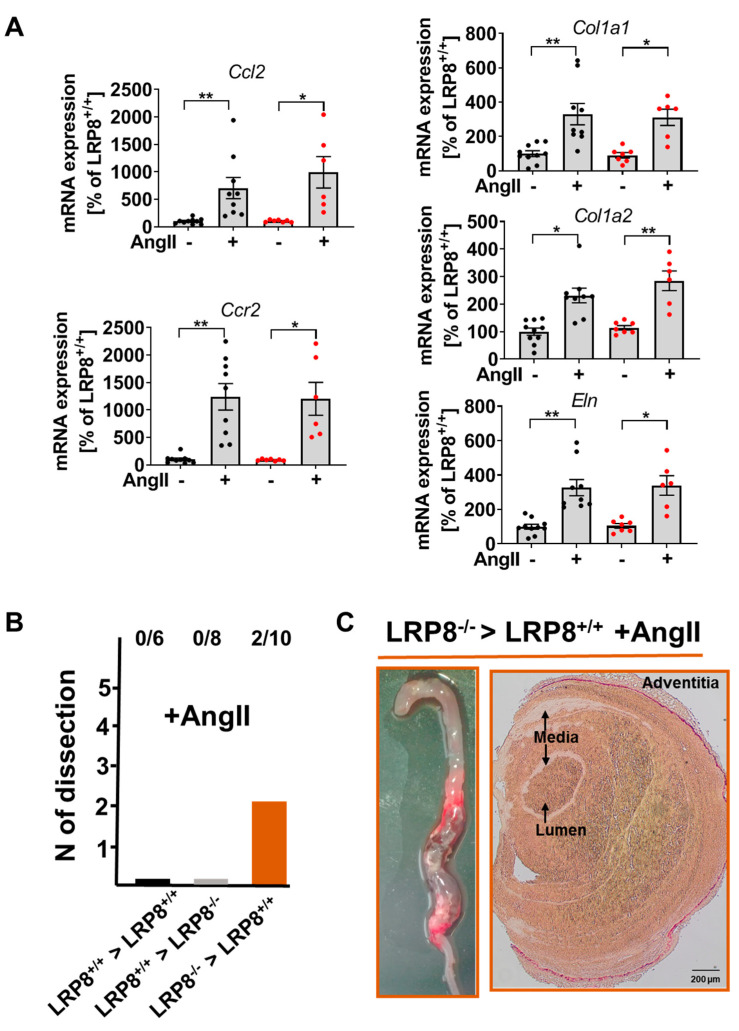
Critical role of myeloid cells to drive aortic dissection in AngII infused LRP8^−/−^ mice. (**A**) Aortic mRNA expression of *Ccl2, Ccr2, Col1a1, Col1a2* and *Eln*. One dot corresponds to one aorta of one animal. *n* = 6–10 animals/group. Data are presented as mean ± SEM; * *p* < 0.05, ** *p* < 0.01; vs. sham treatment of the same strain. One-way ANOVA and Bonferroni’s multiple comparison test. (**B**) Aortic dissection development following bone marrow transfer and AngII infusion (Bone marrow from LRP8^+/+^ to LRP8^+/+^, from LRP8^+/+^ to LRP8^−/−^ and from LRP8^−/−^ to LRP8^+/+^). Six LRP8^+/+^ received LRP8^+/+^ BM, 8 LRP8^−/−^ received LRP8^+/+^ BM and 10 LRP8^+/+^ received LRP8^−/−^ BM. (**C**) Representative images of macroscopic inspection of the aorta as well as sirius red staining of aortic section of LRP8^−/−^ → LRP8^+/+^ bone marrow transfer mice, infused with AngII (scale bar 200 µm).

Expression levels of *Ccl2, Ccr2, Eln, Col1a1* and *Col1a2* mRNA encoding for monocyte chemoattractant protein-1 (MCP-1), the MCP-1 receptor, elastin and collagen (type I, alpha 1 and type I, alpha 2), respectively, were similar in LRP8^+/+^ and LPR8^−/−^ mice, both in response to AngII infusion or sham (Figure 3A). To investigate, whether the vascular phenotype was related to myeloid cells, we performed bone marrow transplantation studies. Interestingly, LRP8^+/+^ → LRP8^−/−^ chimeras were largely protected from AngII-induced aortic dissections, whereas LRP8^−/−^ → LRP8^+/+^ phenocopied the LRP8^−/−^ mice, strongly suggesting that the loss of LRP8 on myeloid cells is largely responsible for the phenotype observed in AngII infused LRP8^−/−^ mice (Figure 3B,C).

## 3. Discussion

We report here the unexpected formation of aortic dissection in LRP8^−/−^ mice in response to AngII infusion. We observed that blood pressure, assessed by tail cuff measurements, was not significantly increased in LRP8 deficient mice infused with AngII, which may be compatible with aortic dissection complicated by malperfusion syndrome or distributive shock with hypotension. 

The absence of LRP8 may lead to the formation of dissections due to alterations of both endothelial and vascular smooth muscle cell layers. Indeed, LRP8 was found to mediate endothelial barrier and antiapoptotic signaling through activated protein C in endothelial cells [10]. More recent data suggested that the lack of LRP8 leads to the acceleration of vascular smooth muscle cell senescence, independently of the effects of Apolipoprotein E (ApoE) promoting vascular smooth muscle hyperplasia [15]. In our study, we showed that the phenotype is driven mainly by LRP8 deficiency on myeloid and not vascular cells, which subsequently triggers disruption of vascular homeostasis.

While AngII infusion in ApoE^−/−^ is known to trigger aortic aneurysm formation as well as aortic dissection due to concomitant vascular wall remodeling and recruitment of monocytes and macrophages, we did not find differences between aortas from LRP8^+/+^ and LRP8^−/−^ mice concerning histology or expression of prominent genes involved in vascular inflammation or remodeling [16]. AngII infusion also leads to aortic dissection in heterozygous collagen type III mutated mice which are a model of vascular Ehlers–Danlos syndrome, a disease associated with early-onset of arterial rupture [17]. However, in this model the increase in blood pressure was the major trigger of vessel injury.

Vascular dysfunction was comparable in AngII-infused LRP8^+/+^ and LRP8^−/−^ mice, with slightly more prominent accumulation of pro-inflammatory Ly6C^hi^ monocytes and Ly6G^+^ neutrophils in the aortas of LRP8^−/−^ +AngII group. Regarding the importance of the receptor in the development of vascular inflammation, we cannot exclude a selection bias due to the death of some LRP8 deficient mice within the first week of infusion. In order to focus on the possible role of immune cells we infused mice with AngII following bone marrow transfer. Interestingly, none of the LRP8^−/−^ mice that received LRP8^+/+^ bone marrow developed aortic dissections, while 2 out of 10 LRP8^+/+^ mice that received LRP8^−/−^ bone marrow did. These results indicate that, despite the fact that irradiation is known to limit aneurysm formation and rupture [18], myeloid immune cells may play a major role in the formation of aortic dissection that was observed in LRP8^−/−^ mice infused with AngII.

In human physiopathology, a direct role of LRP8 in the development of aortic dissection has not yet been demonstrated. Concerning the role of the renin–angiotensin system in the development and rupture of aortic aneurysms, reducing AngII with angiotensin-converting enzyme (ACE) inhibitors was found to be beneficial in limiting aneurysm progression, independently of its role as an antihypertensive drug, since other antihypertensive drugs could not lower the risk of aneurysm rupture [19]. Here, the anti-inflammatory role of ACE inhibitors was the main effector of the protective effect. Inflammatory signals and neutrophil infiltration are associated with the development of aortic dissection both in humans and mice [20,21]. In human aortic samples obtained during surgical repair following aortic dissection, STAT3 activation was associated with neutrophil infiltration and in a mouse model of acute aortic dissection, the CXCL1 (chemokine (C-X-C motif) ligand 1)/granulocyte-colony stimulating factor pathway was highlighted in the recruitment of neutrophils. Very recently, CD44 which is the main receptor for extracellular matrix proteins such as hyaluronan, was found to promote adhesion of leukocytes to endothelial cells and participate in the development of aortic dissection [22]. In this work, lack of CD44 was able to limit neutrophil migration. 

In Marfan’s syndrome, which is characterized by a fibrillin-1 gene mutation and excessive TGF-β leading to the development of aortic arch aneurysm and dissection, it is not clear if targeting the renin–angiotensin system can be beneficial [23]. Despite a positive effect in mouse models, administration of an AngII receptor type 1 blocker (ARB) in Marfan’s syndrome patients was not associated with an improvement of the disease compared to conventional treatment with betablockers. Similarly, despite promising results for ARBs in mouse models as well as in human patients, the benefit of such treatment is still an open question [24].

In conclusion, our results indicate that LRP8 deficient mice may be a new and useful model to study aortic dissection. Trachet et al. had concluded from previous studies, that ApoE^−/−^ mice infused with AngII are more clinically relevant models to study aortic dissections than aortic aneurysms [25]. Contrary to other models of aortic dissections with lower rates of death [26], AngII-infused LRP8 deficient mice have high mortality rates, reflecting the lethality of aortic dissection in human medicine [27].

## 4. Materials and Methods 

### 4.1. Animals, In Vivo Treatment and Blood Pressure Recording 

LRP8^−/−^ (B6;129S-LRP8^tm3Her^/J)(The Jackson Laboratory) and LRP8^+/+^ littermates were infused s.c. with angiotensin II (1 mg⋅kg^−1^⋅d^−1^ for 7 days or 0.7 mg⋅kg^−1^⋅d^−1^ for 28 days) via miniosmotic pumps (model 1007D and 2004, ALZET, Cupertino, CA, USA) vs. sham. Blood pressure measurements were performed by tail cuff using the Coda Monitor System (Kent Scientific, Torrington, CT, USA) 6 days after pump implantation or every week for the 28 days pumps. Male mice (10 to 12 weeks old) were used as experimental animals. All procedures performed on mice were approved by the Institutional Animal Care and Use Committee (Landesuntersuchungsamt Rheinland-Pfalz, Koblenz, Germany; animal experimental approvals G15-1-051 (2015) and G18-1-080 (2018)), following the German Law on the Protection of Animals.

### 4.2. Vascular Reactivity Studies

Vascular reactivity studies (concentration–relaxation curves in response to vasodilators) were performed as described previously [7,8]. To assess vasodilator properties of isolated aortic segments (~4 mm), they were mounted to force transducers in organ chambers to test their response to Ach. The aortic rings were pre-constricted with prostaglandin F_2_α (3 nM) to reach 80% of the tone induced by KCl (80 mM). Concentration–relaxation curves were recorded in response to the endothelium-dependent vasodilator ACh (1 nM–3 µM) and normalized to the preconstriction achieved by prostaglandin F_2_α for each individual ring (% relaxation as a reciprocal of the 100% preconstriction achieved by prostaglandin F_2_α).

### 4.3. Intravital Fluorescence Microscopy

For anesthesia and analgesia, mice received intraperitoneal injections of midazolam (5 mg⋅kg^−1^; Ratiopharm GmbH), medetomidine (0.5 mg⋅kg^−1^ body weight), and fentanyl (0.05 mg⋅kg^−1^ body weight; Janssen-Cilag GmbH, Hilden, Germany). Animals were fixed on a custom built-stage to maintain a physiological temperature. The right and left common carotid arteries were dissected free. For the quantification of leukocyte adhesion, 100 µL acridine orange (0.5 mg⋅mL^−1^, Sigma-Aldrich, Saint-Louis, MO, USA) was injected via a jugular vein catheter (0.28 mm ID, 0.61 mm OD; Smiths Medical Deutschland GmbH, Fraureuth, Germany) to stain circulating leukocytes in vivo. Measurements were performed with a high-speed wide-field Olympus BX51WI fluorescence microscope using a long-distance condenser and a 10 × (NA 0.3) water immersion objective with a monochromator (MT 20E; Olympus Deutschland GmbH) and a charge-coupled device camera (ORCA-R^2^, Hamamatsu Photonics). For image acquisition and analysis, Realtime Imaging System excellence RT (Olympus Deutschland GmbH, Düsseldorf, Germany) software was used. Cell recruitment was quantified in four fields of view (100 × 150 μm) per carotid artery. Adherent cells were defined in each vessel segment as cells that did not move or detach from the endothelial lining within an observation period of 10 s and presented per mm^2^.

### 4.4. Flow Cytometric Analysis of Aortic Lysates

Aortic vessels were cleaned of perivascular fatty tissue and adventitia, minced and digested by 1 mg/mL liberase^TM^ (Roche Diagnostics, Basel, Switzerland) as described [7,28]. Single-cell suspensions were stained with CD45-APCefluor780, CD11b-PE, Ly6G-FITC, Ly6C-PerCP-Cy5.5, NK1.1-PE-Cy7, F4/80-APC, Viability Dye eFluor 506 monoclonal antibodies. At least 2.5 to 4.0 × 10^5^ cells were treated with Fc-block, washed and surface-stained. Based on a live gate, events were acquired and analyzed using a BF FACS CANTO II flow cytometer (Becton Dickinson) and FlowJo, respectively.

### 4.5. Picro-Sirius Red Staining

Aortas were fixed in paraformaldehyde (4%) and embedded in paraffin. Samples were stained in picro-sirius red solution (0.1% with 1.2% picric acid). Finally, specimens were dehydrated with ethanol and coverslipped with Entellan. Images were taken using an Olympus IX73 microscope and Olympus SC30 camera.

### 4.6. mRNA Expression Analysis

mRNA expression analysis was performed as published before [8]. Briefly, aortas were snap-frozen and homogenized with the Tissue Lyser II (Qiagen, Hilden, Germany) and for RNA isolation the modified guanidine isothiocyanate method of Chomczynski and Sacchi [29] was used. RT-PCR was performed with the CFX96 Real-Time PCR Detection System (BioRad, Munich, Germany). For RT-PCR analysis 0.125 µg of total RNA was used with the QuantiTect Probe RT-PCR kit (Qiagen, Hilden, Germany). TaqMan Gene Expression assays were used as probe and primer sets (Applied Biosystems, Foster City, CA) for TATA-box binding protein (mouse: Tbp, Mm00446973_m-1).

*Ccl2* (mouse: Mm00441242_m1), *Ccr2* (mouse: Mm00438270_m1), *Eln* (mouse: Mm00514670_m1), *Col1a1* (mouse: Mm00801666_g1), *Col1a2* (mouse: Mm00483888_m1). Results were quantified with the relative Ct method and normalized to TATA box binding protein as the endogenous control.

### 4.7. Bone Marrow Transplantation

Mice were irradiated with 9.5 Gy (Cs137 exposure by OB58-BA; Buchler) and treated with Borgal antibiotic (Hoechst Roussel Vet, Milton Keynes, UK) orally in drinking water one week before and two weeks after irradiation. Bone marrow isolation from femur and tibia of LRP8^+/+^ and LRP8^−/−^ mice was performed and 5 × 10^6^ cells were transferred into the irradiated LRP8^+/+^ and LRP8^−/−^ recipient animals. Bone marrow transplanted mice were infused with AngII (1 mg⋅kg^−1^⋅d^−1^ for 7 days) after 8 weeks.

### 4.8. Statistical Analysis

Results are presented as mean ± standard error of the mean. Statistical calculations were performed with GraphPad Prism (GraphPad Software Inc). One- or two-way ANOVA with post hoc Bonferroni’s or Dunn’s multiple comparison test were used as appropriate. Kaplan–Meier curves were compared using a log-rank test. *p* values of < 0.05 were considered significant and marked by asterisks (* *p* < 0.05; ** *p* < 0.01).

## Figures and Tables

**Table 1:** Blood count from LRP8^+/+^ and LRP8^−/−^ mice, sham treated or infused with AngII.

	LRP8^+/+^	LRP8^+/+^ + AngII	LRP8^−/−^	LRP8^−/−^ + AngII
*n*	7	6	6	6
WBC (10^3^/µL)	4.0 ± 0.4	4.5 ± 0.6	4.7 ± 0.6	4.6 ± 0.5
RBC (10^6^/µL)	8.0 ± 0.1	8.8 ± 0.3 *	8.3 ± 0.1	9.1 ± 0.4
HGB (g/dL)	12.0 ± 0.1	12.8 ± 0.4	12.0 ± 0.2	13.1 ± 0.5
HCT (%)	42 ± 0.4	46 ± 1.4 *	43 ± 0.6	47 ± 2.1
Platelets (10^3^/µL)	967 ± 23	1035 ± 90	1001 ± 79	929 ± 74
MPV (fl)	5.7 ± 0.1	6.0 ± 0.1 *	5.7 ± 0.1	5.9 ± 0.1 *

Angiotensin II (AngII) was infused 1 mg/kg/d for 7 days. LRP8: lipoprotein receptor-related protein 8; WBC: white blood cells; RBC: red blood cells; HGB: hemoglobin; HCT: hematocrit; MPV: mean platelet volume. Data are presented as mean ± SEM; * *p* < 0.05 vs. no AngII mice of the same strain. One-way ANOVA and Bonferroni’s multiple comparison test.

## Abbreviations

ACh	Acetylcholine
AngII	Angiotensin II
ApoER2	Apolipoprotein E receptor 2
FXI	Factor XI
GPIbα	Glycoprotein Ib alpha
LRP8	Low-density lipoprotein receptor-related protein 8
